# Longitudinal Doppler references for monochorionic twins and comparison with singletons

**DOI:** 10.1371/journal.pone.0226090

**Published:** 2019-12-06

**Authors:** Daniela Casati, Marcella Pellegrino, Ivan Cortinovis, Elena Spada, Mariano Lanna, Stefano Faiola, Irene Cetin, Maria Angela Rustico

**Affiliations:** 1 Fetal Therapy Unit 'Umberto Nicolini', Vittore Buzzi Children's Hospital, University of Milan, Milan, Italy; 2 Department of Obstetrics and Gynecology, Vittore Buzzi Children's Hospital, University of Milan, Milan, Italy; 3 Laboratory G.A. Maccacaro, Department of Clinical Sciences and Community Health, University of Milan Milan, Italy; 4 Neonatal Unit, University of Turin, City of Health and Science of Turin, Turin, Italy; Careggi University Hospital of Florence, ITALY

## Abstract

**Objectives:**

To construct monochorionic (MC) twin-specific longitudinal Doppler references for umbilical artery pulsatility index (UA-PI), middle cerebral artery (MCA) PI and peak systolic velocity (PSV) and ductus venosus (DV) PI derived from a strictly selected cohort of uncomplicated MC twins. The secondary aim of the study was to compare our findings with singleton reference charts.

**Methods:**

A retrospective evaluation was made of all consecutive uncomplicated MC twin pregnancies referred to our Unit from 2010 to 2018. Fortnightly serial examinations were performed of UA-PI, MCA-PI, MCA-PSV and DV-PI, according with the clinical protocol, from 20 to 37 weeks of gestation. We included cases with at least four ultrasound examinations, delivery at our hospital and complete neonatal follow up. A two-step method was used to trace the estimated centile curves: estimation of the median was performed with appropriate fractional polynomials by a multilevel model and estimation of the external centiles through the residuals (quantile regression). The comparison with singletons was made by plotting the references derived from the present study on the referred charts commonly used for singletons.

**Results:**

The study group comprised 150 uncomplicated MC twin pairs. Estimated centiles (3^rd^, 5^th^, 10^th^, 50^th^, 90^th^, 95^th^, 97^th^) of UA-PI, MCA-PI, MCA-PSV and DV-PI in function of the gestational age are presented. The comparison with singletons showed substantial differences, with higher UA-PI and lower MCA-PI and PSV median values in MC twins. Median DV PI values were similar to the values for singletons, while the upper centiles were higher in MC twins.

**Conclusions:**

This study sets out MC twin-specific longitudinal references for UA-PI, MCA-PI, MCA-PSV and DV-PI derived from the largest series of uncomplicated MC twin pregnancies presently available. The comparison with singleton reference values underscores the deviation from physiology that is intrinsic to these unique pregnancies and supports the need for MC twin-specific charts.

## Introduction

Doppler ultrasound investigation of umbilical and fetal circulation is widely used for fetal surveillance in high risk pregnancies, with proven efficacy for identifying fetal compromise and improving pregnancy outcomes [1,2]. The methodology for obtaining fetal Doppler waveforms has been standardized [3] and, as regards singleton pregnancies, several reference charts for Doppler parameters are currently available, derived both from cross-sectional and longitudinal studies, and with a proper sample size [4–9].

As for intrauterine growth charts, some authors have suggested that singleton Doppler nomograms are not appropriate for interpreting findings in twins [10–12]. Additional consideration is necessary for monochorionic (MC) twins who have interdependent circulations deriving from placental vascular anastomoses which may give rise to specific Doppler waveforms. In comparison to both dichorionic (DC) twins and singleton pregnancies, MC pregnancies are at higher risk of severe complications such as intrauterine growth restriction (IUGR), intrauterine fetal demise, severe congenital anomalies, neurological impairment, perinatal and neonatal morbidity and mortality [13–18]. Moreover, since conditions such as twin-to-twin transfusion syndrome (TTTS) and selective IUGR (sIUGR) have a significant impact on fetal haemodynamics and Doppler waveforms, the Doppler examination plays a major role in the surveillance and management of MC pregnancies. In 2014, a prospective multicenter cohort study in Ireland reported the longitudinal references for umbilical artery (UA) pulsatility index (PI) and resistance index (RI), middle cerebral artery (MCA) PI and peak systolic velocity (PSV) and cerebroplacental ratios (CPR) derived from 508 DC and 110 MC twin pregnancies from 24 to 38 weeks of gestation. They found that both in DC and MC twins, UA-PI and UA-RI appeared to be higher than in singletons, while MCA-PI, MCA-PSV and CPR appeared lower [12].

Longitudinal observations make it possible to assess valid reference ranges and compute conditional centiles which cannot be derived from cross-sectional data; however, longitudinal observations in twin pregnancies represent data which are highly correlated (being between-subject as well as within-subject) and such data require complex statistical analyses [12,19–21].

The aim of the present study was to construct MC twin-specific longitudinal Doppler references for UA- PI, MCA- PI and PSV and ductus venosus (DV) PI derived from a carefully selected cohort of uncomplicated monochorionic twins. The secondary aim was to compare our findings with the reference values for singletons.

## Methods

### Study population

In this retrospective study, we analysed the data deriving from all consecutive uncomplicated MC twin pregnancies referred to the ‘Umberto Nicolini’ Fetal Therapy Unit of the V. Buzzi Children’s Hospital, University of Milan, Italy, between January 2010 and August 2018. The inclusion criteria were as follows: a MC twin pregnancy; a minimum of four ultrasound (US) examinations performed at our Unit from 20 to 37 weeks of gestation; delivery at V. Buzzi Children’s Hospital at an appropriate gestational age (GA); good outcome at birth, and availability of a complete neonatal follow up. Exclusion criteria were fetal and maternal complications that can have an impact on Doppler waveforms. In particular, we excluded MC pregnancies complicated by sIUGR, TTTS, twin anemia-polycythemia sequence (TAPS), major anatomical and/or genetic anomalies, discrepancy of amniotic fluid (defined as discrepancy of the deepest vertical pocket [DVP] greater than 4 centimetres), discrepancy of fetal/neonatal weight> 20%, neonatal weight less than the 5^th^ centile of either twin (according to INeS references for firstborn neonates [22]), fetal death of one or more twins, as well as pregnancies complicated by hypertensive disorders and severe preterm delivery.

### Data collection

All patients meeting the inclusion criteria had been diagnosed as having a MC twin pregnancy at first-trimester US examination: this was confirmed after birth with macroscopic analysis of the placenta and membranes performed by a specialist in feto-maternal medicine and by histopathological examination. Pregnancies were dated according to crown-rump length (CRL) measurement in the first trimester [23]. At referral, both twins underwent detailed evaluation of fetal anatomy and biometry, amniotic fluid DVP, Doppler evaluation of UA, MCA and DV, placental location and cords insertions. MC twin estimated fetal weight (EFW) was obtained according to the formula described by Ananth *et al*.[24] and intertwin EFW discordance was calculated using the formula: *(large twin EFW–small twin EFW) x100/large twin EFW* [3]. At the first US assessment, and at each following US examination, Twin 1 and Twin 2 were labelled according to laterality (left/right) or vertical orientation (top/bottom) and cord insertion was mapped so that each twin was followed longitudinally. In the course of the final ultrasound examination, we identified the twin closer to the cervix, in order to distinguish Twin 1 and Twin 2 after birth.

All uncomplicated MC pregnancies were monitored longitudinally every 2 weeks, from the 16th week until delivery as per clinical protocol by dedicated sonographers (M.A.R., M.L., S.F., D.C.) using a GE Voluson 730 Expert or E8 Ultrasound machine (GE Medical Systems, Zipf, Austria), equipped with a 4–8 MHz probe. Doppler waveforms were acquired during fetal quiescence in accordance with the ISUOG practice guidelines specific for each vessel [3]. All ultrasonographic data were automatically transferred to a software system (Viewpoint © 5.6.21.12, General Electric Healthcare). The stored data were retrospectively evaluated by a single operator (M.P.) and only those cases with at least five reproducible waveforms which conformed to ISUOG quality recommendations were considered eligible for the analysis.

Data on pregnancy and neonatal outcome were collected from hospital records. These comprised maternal characteristics (age, BMI, ethnicity, parity, pregnancy onset-spontaneous versus medical assisted), mode of delivery, GA at birth, fetal sex, birthweight, admission to the neonatal intensive care unit (NICU) and neonatal follow up. Neonates/infants were screened as per clinical protocol with serial neurological examinations, abdominal US scan at 2 months, brain magnetic resonance imaging during spontaneous sleep at 1 month, SIDS (Sudden Infant Death Syndrome) screening (electrocardiography, echocardiography and cardiological examination) at 1 month.

All data were obtained from medical records in a fully anonymized and de-identified manner, and none of the authors had access to identifying information. The study complied with our Institution’s research guidelines for clinical observational and retrospective studies.

### Statistical analysis

For the construction of the charts, Doppler variables were transformed where necessary so as to normalize them. In a first step, the median (50^th^ centile) according to GA was traced using fractional polynomials with a multilevel model, taking into account the effect of the mothers (inter-subjects variability) and of the fetuses into same mother (inter-twin variability). As suggested by Royston and Altman [25], the best fractional polynomial was chosen for each variable using a dual criterion: (1) the maximum value of the G function, and (2) the plausibility of the resulting shape. Given the complex shape of the variables analysed, fractional polynomials up to 3 elements (trinomial) were considered.

As a second step, we explored various different ways of estimating the external centiles (3^rd^, 5^th^, 10^th^, 25^th^, 75^th^, 90^th^, 95^th^ and 97^th^): the analysis of absolute residuals [26], the computation of the variance by GA as a sum of its components estimated from the multilevel analysis [5], and the quantile regression on the residuals [27]. The latter method proved to be the most reliable for the description of the data.

Reference charts were not traced separately by maternal characteristics, such as maternal age, body mass index, ethnicity, and parity, in order to obtain a useful and easy tool for clinicians.

All the analyses were performed using SAS software version 9.4 [SAS Institute. Inc. Cary, NC, USA. 2004]. In particular, the medians were estimated using the PROC MIXED in SAS software version 9.4 [28,29], while the external centile using PROC QUANTREG [27].

For the comparison with singleton nomograms, we decided to use the works by Acharya *et al*.[5] for UA- PI, Ebbing *et al*. [6] for MCA- PI and PSV, and Kessler *et al*. [7] for DV- PI given their high methodological quality [4] and the similar method used for data collection (longitudinal).

## Results

### Population

**Fig 1:**
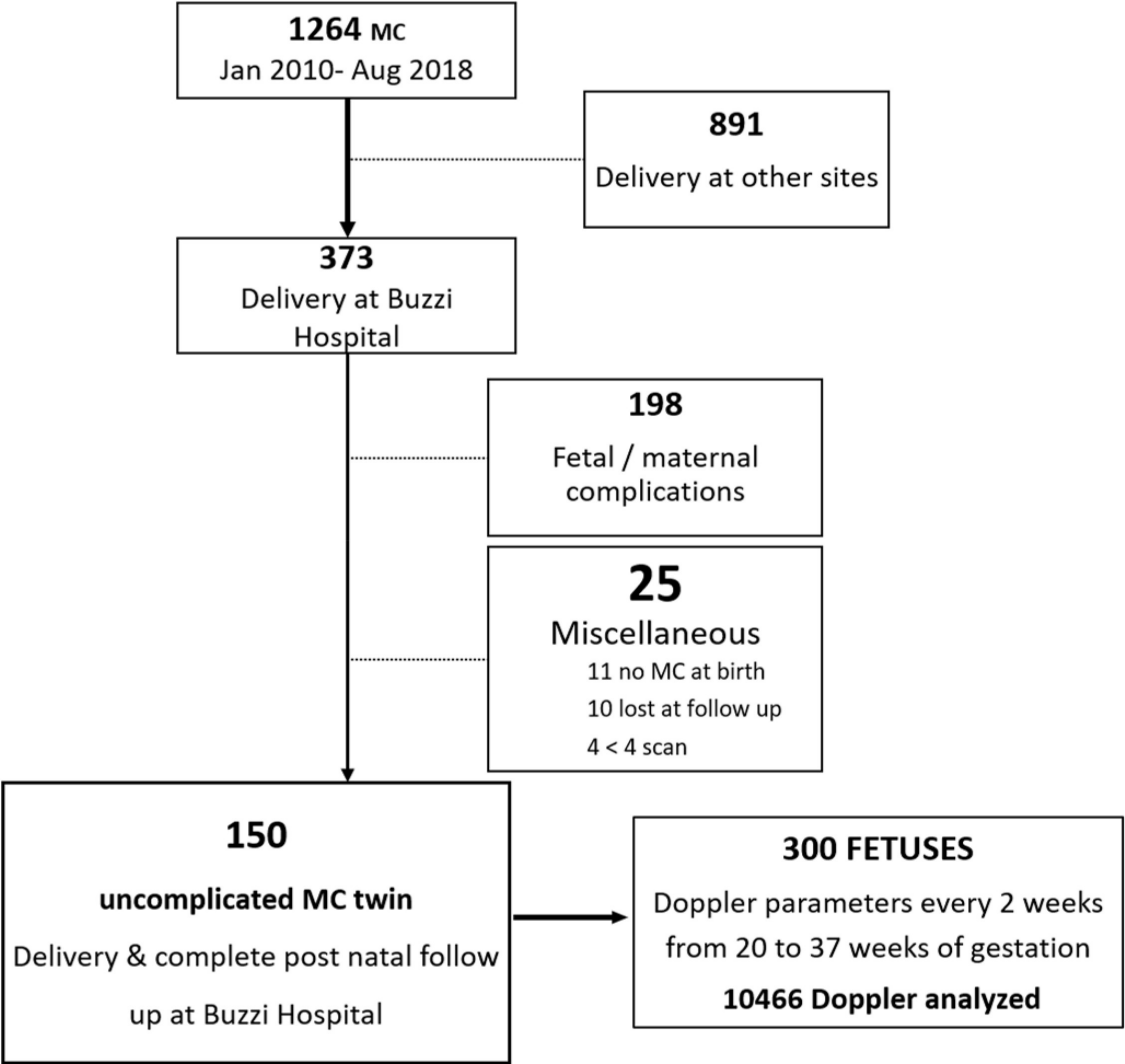
Population flow chart. MC: monochorionic.

During the study period, 1264 MC twin pregnancies were referred to our Unit. We excluded 891 women who delivered in other hospitals, 198 cases for pregnancy complications (either fetal or maternal), 11 cases which were not confirmed as monochorionic after birth, 10 cases lost at follow-up, and a further 4 cases for having fewer than 4 examinations performed at our Unit. The final study group thus consisted of 150 MC twin pairs (300 fetuses) with a median of 8 (range 4–9) ultrasound scans performed from 20 to 37 weeks and a total of 10466 Doppler parameters analysed (see population flow chart, Fig 1). The characteristics and outcome of the study population are summarised in Table 1.

**Table 1:** Characteristics and outcome of the study population.

*Variable*	*Uncomplicated MC twin pregnancies*
**Maternal characteristics**	
Maternal age (years); median (IQR)	33 (30–36)
Ethnicity	
– *Caucasian*	129 (86.0)
– *Afro-Caribbean*	13(8.6)
– *East Asian*	8 (5.4)
BMI (Kg/m^2^); mean (SD)	21.5 (19.7–23.8)
Nulliparous; n (%)	82 (54.6)
ART pregnancy; n (%)	10 (6.6)
Type of MC pregnancy; n (%)	
– *MC diamniotic*	140 (93.4)
– *MC monoamniotic*	7 (4.6)
– *BC triamniotic*	3 (2.0)
GA at delivery (weeks+days); median (IQR)	36+0 (35+4–36+2)
Cesarean section; n (%)	149 (99.3)
**Neonatal characteristics**	
Male pairs, n (%)	67 (44.7)
Birthweight (grams); mean (SD)	2310 (313)
Birthweight (z-score)^a^; mean (SD)	-0.54 (0.68)
Inter-twin birthweight discrepancy (%); median (IQR)	7.5 (4.0–11.0)
Apgar score 1’; median (IQR)	9 (8–9)
Umbilical artery pH; median (IQR)	7.34 (7.31–7.36)
Hb at birth (g/dl); median (IQR)	16.1 (14.85–16.1)
Inter-twin Hb discrepancy(g/dl), median (IQR)	1.0 (0.5–1.7)
Admission to NICU; n (%)	63 (21)

MC: monochorionic; BMI: body mass index; ART: assisted reproductive technology; GA: gestational age; Hb: haemoglobin; NICU: neonatal intensive care unit.

^a^ according to INeS reference for firstborn neonates [22]

Postnatal follow up ranged from 6 months to 8 years (median 48 months) and was recorded for all neonates/infants through consultation of medical reports. 63 out of 300 (21%) newborns were admitted to NICU, mainly for the need of ventilatory support, with a median length of hospitalization of 13 days (10–19). No major structural abnormalities or neurologic morbidity were found.

### MC twins’ references and comparison with singletons

Table 2 reports the transformation applied to normalize each variable, the fractional polynomial chosen to estimate the median, and the interclass correlation coefficient (ICC) indicating how much of the total variation is accounted for by the mothers and by the fetuses into same mother [29].

**Table 2:** Transformation applied to normalize the variable, fractional polynomial chosen to estimate the median, and Interclass Correlation Coefficient (ICC).

Variable	Variable normalization	Fractional polynomial (exponents)	ICC	
			Mother	fetus (mother)
UA-PI	Logarithmic	monomial (0)	0.249	0.073
MCA-PI	Sqare root	trinomial (-0.5;0;+0.5)	0.132	0.026
MCA PVS	No trasformation	trinomial (+0.5;+1;+2)	0.154	0.048
DV-PI	Cubic root	binomial (-0.5; 0)	0.079	0.051

UA-PI: umbilical artery pulsatility index; MCA-PI: middle cerebral artery pulsatility index; MCA-PSV: middle cerebral artery peak systolic velocity; DV-PI: ductus venosus pulsatility index.

The UA-PI median was estimated with a monomial fractional function, while MCA-PI and PVS medians were estimated using a trinomial fractional function; a binomial one was used for the DV-PI median. The total variation accounted for by the mother ranged from 8% (DV-PI) to 25% (UA-PI), and by the fetuses into same mother from 2.6% (MCA-PI) to 7.3% (UA-PI) (Table 2).

**Fig 2:**
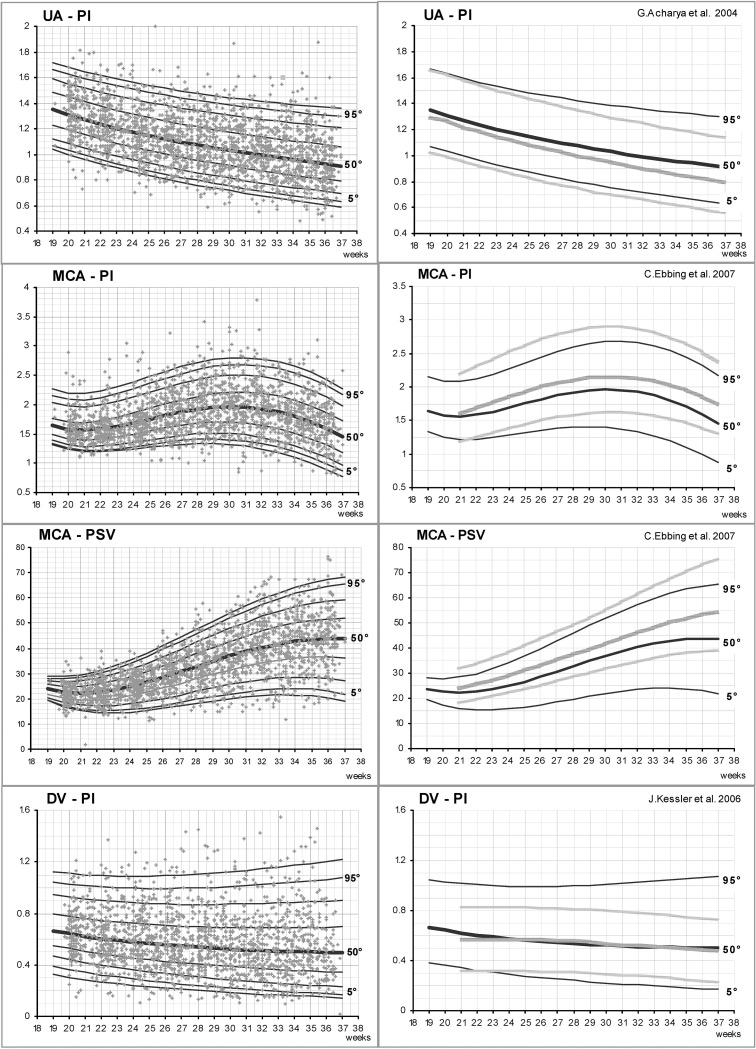
*Left panels*: umbilical artery pulsatility index (UA-PI), middle cerebral artery pulsatility index (MCA-PI), middle cerebral artery peak systolic velocity (MCA-PSV) and ductus venosus pulsatility index (DV-PI) values in 300 uncomplicated monochorionic twin fetuses between 20 and 37 weeks of gestation; the lines indicate the estimated 3^rd^, 5^th^, 10^th^, 50^th^, 90^th^, 95^th^,97^th^ centiles. *Right panels*: comparison of the 5^th^, 50^th^ and 95^th^ estimated centiles curves of MC twins (present study, black lines) and singleton reference values by Acharya *et al*.[5] for UA-PI, Ebbing *et al*.[6] for MCA-PI and PSV, and Kessler *et al*.[7] for DV-PI (grey lines).

Fig 2 reports the median (50^th^ centile) and the 3^th^, 5^th^, 10^th^, 90^th^, 95^th^ and 97^th^ estimated centiles curves for the four Doppler parameters under investigation (left panels) and the comparison between the 5^th^, 50^th^ and 95^th^ estimated centile curves of singletons (data from literature) [5–7] and of MC twins (present study) (right panels).

Tables 3–6 report the GA-specific values for the 3^rd^, 5^th^, 10^th^, 50^th^, 90^th^, 95^th^, and 97^th^ centiles of each Doppler variable under study.

**Table 3:** Gestational age (GA)-specific longitudinal reference centiles for umbilical artery pulsatility index (UA-PI).

GA (exact week)	UA-PI centile								
	3^rd^	5^th^	10^th^	25^th^	50^th^	75^th^	90^th^	95^th^	97^th^
20	1.00	1.03	1.09	1.19	1.31	1.45	1.55	1.63	1.68
21	0.96	1.00	1.05	1.15	1.27	1.41	1.52	1.59	1.65
22	0.93	0.96	1.02	1.12	1.24	1.38	1.49	1.56	1.62
23	0.90	0.93	0.99	1.09	1.20	1.35	1.46	1.54	1.59
24	0.86	0.90	0.96	1.06	1.17	1.32	1.43	1.51	1.56
25	0.84	0.87	0.93	1.03	1.15	1.29	1.41	1.49	1.54
26	0.81	0.84	0.90	1.00	1.12	1.26	1.38	1.46	1.52
27	0.78	0.82	0.88	0.98	1.10	1.24	1.36	1.44	1.50
28	0.76	0.80	0.86	0.96	1.07	1.22	1.34	1.42	1.48
29	0.74	0.77	0.84	0.93	1.05	1.20	1.32	1.41	1.46
30	0.72	0.75	0.81	0.91	1.03	1.18	1.31	1.39	1.44
31	0.70	0.73	0.80	0.89	1.01	1.16	1.29	1.37	1.43
32	0.68	0.71	0.78	0.87	0.99	1.14	1.28	1.36	1.41
33	0.66	0.70	0.76	0.86	0.97	1.12	1.26	1.35	1.40
34	0.64	0.68	0.74	0.84	0.96	1.10	1.25	1.33	1.39
35	0.62	0.66	0.73	0.82	0.94	1.09	1.23	1.32	1.38
36	0.61	0.65	0.71	0.81	0.93	1.07	1.22	1.31	1.37
37	0.59	0.63	0.70	0.79	0.91	1.06	1.21	1.30	1.36

**Table 4:** Gestational age (GA)-specific longitudinal reference centiles for middle cerebral artery pulsatility index (MCA-PI).

GA (exact week)	MCA-PI Centile								
	3^rd^	5^th^	10^th^	25^th^	50^th^	75^th^	90^th^	95^th^	97^th^
20	1.23	1.25	1.32	1.42	1.57	1.70	1.97	2.09	2.20
21	1.20	1.22	1.29	1.40	1.56	1.70	1.97	2.08	2.19
22	1.19	1.22	1.29	1.41	1.58	1.73	2.00	2.12	2.23
23	1.21	1.25	1.32	1.45	1.63	1.79	2.06	2.19	2.30
24	1.24	1.28	1.36	1.50	1.69	1.86	2.14	2.27	2.39
25	1.27	1.32	1.40	1.55	1.75	1.94	2.22	2.36	2.48
26	1.30	1.35	1.44	1.60	1.82	2.01	2.30	2.45	2.57
27	1.32	1.39	1.48	1.65	1.88	2.08	2.37	2.53	2.65
28	1.34	1.41	1.50	1.68	1.92	2.14	2.43	2.60	2.72
29	1.34	1.41	1.51	1.70	1.95	2.19	2.48	2.65	2.77
30	1.33	1.40	1.51	1.71	1.96	2.21	2.50	2.68	2.80
31	1.30	1.38	1.49	1.69	1.96	2.21	2.50	2.69	2.80
32	1.25	1.33	1.44	1.65	1.93	2.19	2.48	2.67	2.78
33	1.18	1.27	1.38	1.60	1.87	2.15	2.43	2.62	2.73
34	1.10	1.19	1.30	1.52	1.80	2.08	2.35	2.55	2.66
35	1.00	1.10	1.21	1.42	1.70	1.98	2.25	2.45	2.55
36	0.90	0.99	1.10	1.31	1.59	1.87	2.13	2.32	2.43
37	0.78	0.87	0.97	1.18	1.45	1.73	1.98	2.17	2.27

**Table 5:** Gestational age (GA)-specific longitudinal reference centiles for middle cerebral artery peak systolic velocity (MCA-PSV).

GA (exact week)	MCA-PSV Centile								
	3^rd^	5^th^	10^th^	25^th^	50^th^	75^th^	90^th^	95^th^	97^th^
20	16.90	17.30	18.54	20.15	22.56	25.03	26.71	27.85	28.74
21	15.40	15.95	17.44	19.46	22.19	24.99	27.01	28.45	29.45
22	14.62	15.33	17.06	19.50	22.53	25.67	28.04	29.77	30.87
23	14.42	15.28	17.25	20.11	23.45	26.93	29.64	31.67	32.88
24	14.67	15.68	17.90	21.17	24.83	28.64	31.69	34.02	35.33
25	15.25	16.42	18.88	22.57	26.54	30.69	34.08	36.70	38.12
26	16.07	17.40	20.10	24.20	28.48	32.97	36.71	39.62	41.15
27	17.03	18.51	21.45	25.98	30.57	35.39	39.47	42.68	44.32
28	18.05	19.68	22.87	27.81	32.71	37.87	42.29	45.79	47.54
29	19.04	20.83	24.26	29.61	34.83	40.33	45.09	48.89	50.74
30	19.95	21.89	25.56	31.33	36.86	42.70	47.80	51.89	53.85
31	20.70	22.79	26.71	32.90	38.73	44.91	50.36	54.74	56.81
32	21.23	23.48	27.64	34.24	40.39	46.91	52.70	57.37	59.55
33	21.50	23.90	28.30	35.32	41.78	48.63	54.77	59.74	62.02
34	21.44	24.00	28.64	36.08	42.85	50.04	56.51	61.78	64.17
35	21.02	23.73	28.62	36.47	43.55	51.08	57.90	63.46	65.95
36	20.18	23.05	28.18	36.45	43.84	51.71	58.87	64.72	67.32
37	18.90	21.92	27.29	35.98	43.68	51.88	59.39	65.53	68.24

**Table 6:** Gestational age (GA)-specific longitudinal reference centiles for ductus venosus pulsatility index (DV-PI).

GA (exact week)	DV-PI Centile								
	3^rd^	5^th^	10^th^	25^th^	50^th^	75^th^	90^th^	95^th^	97^th^
20	0.30	0.36	0.44	0.53	0.64	0.78	0.93	1.03	1.11
21	0.29	0.34	0.42	0.51	0.62	0.76	0.91	1.01	1.10
22	0.27	0.32	0.40	0.49	0.60	0.74	0.90	1.01	1.09
23	0.26	0.31	0.38	0.47	0.59	0.73	0.89	1.00	1.09
24	0.24	0.29	0.36	0.45	0.58	0.72	0.88	1.00	1.09
25	0.23	0.28	0.35	0.44	0.56	0.71	0.88	0.99	1.09
26	0.22	0.26	0.33	0.43	0.55	0.71	0.87	0.99	1.09
27	0.21	0.25	0.32	0.42	0.54	0.70	0.87	1.00	1.10
28	0.20	0.24	0.31	0.41	0.54	0.70	0.87	1.00	1.11
29	0.19	0.23	0.30	0.40	0.53	0.69	0.87	1.00	1.11
30	0.19	0.22	0.29	0.39	0.52	0.69	0.87	1.01	1.12
31	0.18	0.21	0.28	0.38	0.52	0.69	0.87	1.02	1.13
32	0.17	0.21	0.27	0.37	0.51	0.69	0.88	1.02	1.15
33	0.17	0.20	0.26	0.37	0.51	0.69	0.88	1.03	1.16
34	0.16	0.19	0.25	0.36	0.51	0.69	0.89	1.04	1.17
35	0.16	0.18	0.24	0.35	0.50	0.69	0.89	1.05	1.19
36	0.15	0.18	0.24	0.35	0.50	0.69	0.90	1.06	1.20
37	0.15	0.17	0.23	0.34	0.50	0.69	0.90	1.08	1.22

### Umbilical artery

It will be observed that UA- PI values decrease with GA (*e*.*g*. the median decreases by almost 30%, from a value of 1.31 at 20 weeks to 0.91 at 37 weeks), while the variability increases with increasing GA (Fig 2, first row, left panel; Table 3). Comparison with the centile curves estimated by Acharya *et al*. [5] shows that the 5^th^, 50^th^ and 95^th^ centiles for MC twins (present study) are higher than those for singletons at every GA considered, and that these differences increase with increasing GA (Fig 2, first row, right panel). For instance, the difference for the 95^th^ centile ranges from 0.01 at 20 weeks (1.63 in the present study, 1.62 in the study by Acharya *et al*.[5]) to 0.16 at 37 week (1.30 in the present study, 1.14 in the study by Acharya *et al*. [5]).

### Middle cerebral artery

MCA-PI values increase with GA until approximately 30–31 weeks and decrease afterwards (Fig 2, second line, left panel; Table 4). MCA-PSV median values increase until approximately 33 weeks, and then decrease (Fig 2, third line, left panel; Table 5). In both cases, the median values found in the present study are considerably lower than the values described by Ebbing *et al*. [6] for singletons. Moreover, there is a noticeable increase in data dispersion and a major difference with singleton reference curves with advancing GA (Fig 2, second and third lines, right panels).

Looking at the MCA-PI 50^th^ centile values, the difference between MC twins and singletons median values ranges from 0.04 at 21 weeks (1.56 in the present study, 1.60 in the study by Ebbing *et al*. [6]) to 0.30 at 37 week (1.45 in the present study, 1.75 in the study by Ebbing *et al*. [6]). Similarly, for the MCA-PSV 50^th^ centile values, the difference ranges from 1.91 cm/sec at 21 weeks (22.18 cm/sec in the present study, 24.09 cm/sec in the study by Ebbing *et al*. [6]) to 10.88 cm/sec at 37 week (43.68 in the present study, 54.56 in the study by Ebbing *et al*. [6]).

### Ductus venosus

DV-PI median values decrease slightly throughout pregnancy. The dispersion grows wider with advancing GA and in particular, the upper centiles are farther from the median compared to the lower centiles (asymmetrical distribution) (Fig 2, fourth line, left panel; Table 6). The comparison with singleton values reported by Kessler *et al*. [7] shows similar values as regards the 50^th^ centile, while the differences in the external centiles (especially the upper ones) increase with increasing GA, given that the estimated curves for the external centiles in singletons show a symmetrical distribution [7] (Fig 2 fourth line, right panel).

As a general consideration, it should be noted that the shapes of the estimated centiles curves for all Doppler parameters are similar between singletons and MC twins.

**Fig 3:**
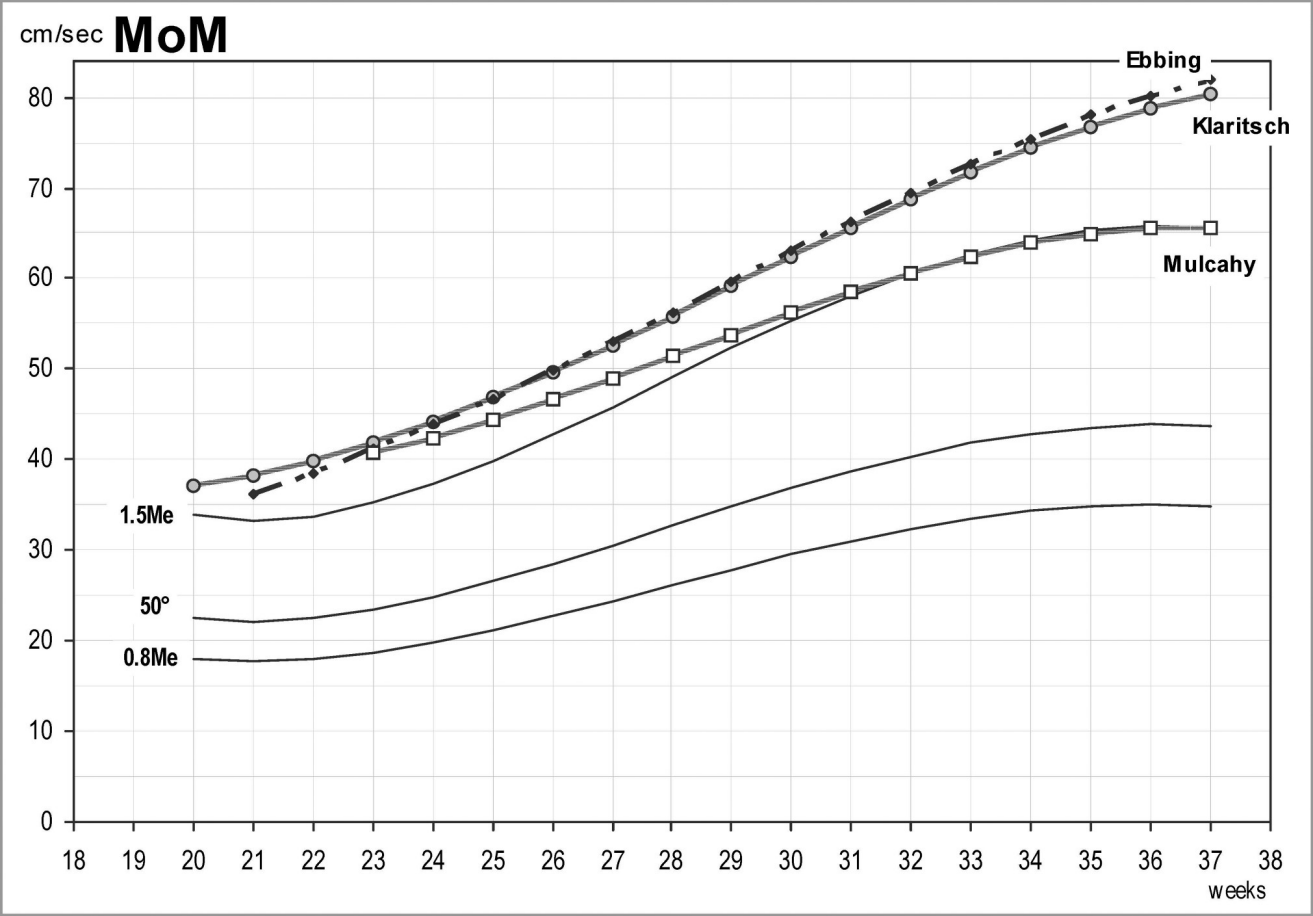
Middle cerebral artery peak systolic velocity (MCA-PSV) multiples of the median (0.8, 1.0, 1.5) derived from the present study (continuous lines), and comparison of MCA-PSV 1.5 MoM derived from the present study and from the MC twin studies of Klarisch *et al*. [21] and Mulcahy *et al*. [12], and the singleton cohort of Ebbing *et al*. [6].

Table 7 reports MCA-PSV MoM (0.8, 1.0, 1.5, 1.7), while Fig 3 shows the comparison among MCA- PSV 1.5 MoM derived from the present cohort, the MC twin cohorts of Klarisch *et al*.[21] and Mulcahy *et al*.[12], and the singleton cohort of Ebbing *et al*. [6]. As can be seen in Table 7, the 1.5 MoM in the present study are similar to those in the study by Mulchay *et al*. [12], particularly for GA over 30 weeks, and considerably lower than the former normative values for singletons6 and MC twins [21], which show similar values to each other. Furthermore, the difference between our 1.5 MoM and the normative one in singletons [6] increases with advancing GA, being 2.86 cm/sec at 21 weeks and rising to 16.32 cm/sec at 37 weeks (Fig 3).

**Table 7:** Middle cerebral artery peak systolic velocity multiples of the median (MCA-PSV MoM) (0.8, 1.0, 1.5, 1.7) derived from 300 uncomplicated monochorionic twin foetuses from 20 to 37 weeks of gestation.

*GA* *(exact week)*	*Middle Cerebral Artery peak systolic velocity (cm/s)*			
	*0*.*8 MoM*	*1*.*0 MoM*	*1*.*5 MoM*	*1*.*7 MoM*
**20**	18.05	22.56	33.84	38.35
**21**	17.74	22.18	33.27	37.71
**22**	18.02	22.53	33.80	38.30
**23**	18.76	23.45	35.18	39.86
**24**	19.86	24.83	37.25	42.21
**25**	21.23	26.54	39.81	45.12
**26**	22.78	28.48	42.72	48.42
**27**	24.45	30.56	45.84	51.95
**28**	26.17	32.71	49.07	55.61
**29**	27.86	34.83	52.25	59.21
**30**	29.49	36.86	55.29	62.66
**31**	30.98	38.73	58.10	65.84
**32**	32.31	40.39	60.59	68.66
**33**	33.42	41.78	62.67	71.03
**34**	34.28	42.85	64.28	72.84
**35**	34.84	43.55	65.33	74.03
**36**	35.07	43.84	65.76	74.53
**37**	34.94	43.68	65.52	74.26

## Discussion

Fetal Doppler velocimetry can be evaluated in both a qualitative and a quantitative manner. Doppler assessment in MC twins has proved useful in diagnosing and managing fetal anemia, selective IUGR and TTTS [30–32]. For these latter conditions, qualitative waveform assessment is mostly used, *e*.*g*. absent or reverse UA or DV *a*-wave end-diastolic flow. Quantitative reference values are lacking for UA, MCA and DV Doppler velocimetry in MC twins, making it more difficult to interpret ominous quantitative findings such as an increased UA-PI value referred to singleton nomograms.

This study has traced longitudinal references for UA-PI, MCA-PI, MCA-PSV and DV-PI for uncomplicated monochorionic twin pregnancies from 20 to 37 weeks of gestation. UA-PI median values decrease with advancing gestational age, MCA-PI and PSV values gradually increase, while DV-PI values slightly decrease throughout pregnancy. For all the parameters, a greater dispersion of values can be observed with advancing gestational age.

To trace the estimated centile curves, a two-phase method was used: in the first step, the median was estimated by a multilevel model using an appropriate fractional polynomial; in the second step, the external centiles were estimated through the residuals using the quantile regression. This method was adopted because it was the most reliable in describing the data. In fact, the percentage of observed values below the 5^th^ and 95^th^ centiles were very close to those expected (from 5.08% to 5.09% below the 5^th^ centile and from 95.02% to 95.08% below the 95^th^ centile (S1 Table).

We compared our findings with the most commonly referred charts used for singletons by plotting our data on the reported median values of Acharya *et al*. [5] for UA-PI, Ebbing *et al*.[6] for MCA-PI and PSV, and Kessler *et al*.[7] for DV-PI.

For every parameter analysed, the shapes of the 5^th^, 50^th^ and 95^th^ percentile curves in our Doppler charts were similar to the corresponding references for singletons (with the exception of the 95^th^ centiles of DV-PI), meaning that both uncomplicated MC twins and singleton fetuses show similar haemodynamic modifications throughout pregnancy. The quantitative comparison, on the other hand, revealed substantial differences in the values for MC twins and singletons, and these differences became more pronounced with increasing gestational age. Indeed, the median values of UA-PI were higher in uncomplicated MC twins than in the singleton cohort reported by Acharya *et al*.[5], while MCA-PI and PSV median values were found to be lower in MC twins at every gestational age when compared to the data from Ebbing *et al*. [6].

The few previous studies comparing MC Doppler data with those of singletons have produced conflicting results, but they suffered from limitations such as small sample size, defective study design and the use of very narrow gestational age ranges with cross-sectional data collection. The parameter most often investigated in MC twins is the MCA-PSV, for which some comparison is available with DC twins and singletons. In a study by Dashe *et al*. [33], no significant differences were found at the 28–32 weeks interval between MCA-PSV values in singletons and in DC twins (36 pairs) and MC twins (16 pairs). Klaritsch *et al*. [21] reported similar results in a longitudinal study comparing MCA-PSV in a cohort of 50 uncomplicated MC twin pregnancies with singleton normative values.

As shown in Fig 3, the MCA-PSV values in the present study were lower compared to the references published so far both for singletons [6,7] and for MC twins[21,33]. By contrast, our findings on MCA-PSV, as well as on UA-PI and MCA-PI are in line with the observations by Mulcahy *et al*. [12] who used a similar study design and a larger sample size compared to former studies. In fact, the study design and the small sample size of previous investigations might have failed to capture the variability in MCA-PSV values that we observed in a larger sample, similarly to what was reported by Mulcahy and colleagues [12].

MCA-PSV MoMs are commonly used to diagnose fetal anemia and the condition of TAPS [8,32]. When applying these new reference ranges in clinical practice, however, we must be careful not to overestimate the incidence of fetal anemia. It should be borne in mind that transient alterations of MCA-PSV may occur [34], and that in an otherwise uncomplicated MC pregnancy, these data need to be interpreted in a longitudinal manner. Moreover, novel diagnostic criteria for TAPS has recently been introduced, and the 1.5 MoM value is no longer the diagnostic cut off of choice, since the inter-twin discrepancy in MCA-PSV MoM seems to perform better [35].

This is the first time that DV-PI has been reported in a cohort of uncomplicated MC twins. The slow decrease in median values is similar to that observed in singletons. As regards the quantitative comparison, we found similar median values to Kessler *et al*. [7]. As for differences in the 5^th^ and 95^th^ estimated centiles, we obtained broader ranges that describe the greater and asymmetrical dispersion of the data in the present twin population. Two factors may have contributed to these differences: one concerning the statistical method and one the physiopathology of MC twins. In fact, to trace our charts, and in particular the external centiles, we did not assume a normal or symmetrical distribution, and the results respect the real dispersion of the data. The same was not done by Kessler and colleagues [7]. In addition, elevated DV PI values could be common in MC twins, possibly due to higher cardiac afterload given by the monochorionic placenta and the continuous intertwin blood exchange.

The higher UA-PI and lower MCA-PI and MCA-PSV median values, and the higher DV PI upper centiles that we found in uncomplicated MC twins mirror the lower growth trajectories observed in these fetuses compared to singleton pregnancies [36,37]. These deviations from the physiological potential of singleton fetuses can be interpreted as adaptative responses to the unique condition represented by monochorionic placenta. Thus, we report the estimated centile curves specific for MC twins as *references* rather than ‘nomograms’ to underline the deviation from physiology that is intrinsic to these pregnancies, even when the course is uneventful.

One strength of this study is that it was performed in a single tertiary care centre with vast experience in the pre- and post-natal management of MC twins. Furthermore, to the best of our knowledge, it is the largest series available based on scrupulous selection criteria and pregnancy monitoring (with a minimum of 4 and a median of 8 examinations for each participant), as well as complete neonatal outcomes. Ultimately, while cross-sectional studies are appropriate for single observations, longitudinally collected data are necessary to construct references for serial measurements [20], as is needed for effective monitoring of MC twins.

One limitation of the study is that the references are traced for the 20–37 weeks interval, leaving out the early second trimester period (16–19 weeks). The retrospective design of the study might represent another limitation (according to what suggested by Oros and colleagues [4]), mainly because of the risk of over-representing at-risk cases and for the quality of the data collected. We think we managed to avoid these potential limitations *a)* by carefully selecting uncomplicated cases and *b)* by ensuring that ISUOG standards were respected in all the ultrasound data which were recorded, as per routine practice at our Unit and thanks also to further qualitative checks performed retrospectively for inclusion in the study.

## Conclusions

This study presents new longitudinal references for UA-PI, MCA-PI, MCA- PSV and DV- PI for MC twins. The substantial differences between singleton and MC twin Doppler values have practical clinical implications and underline the utmost importance of using MC twin-specific charts in daily practice to enable proper diagnosis and management of MC twin complications such as sIUGR, TTTS and TAPS. Further studies would be valuable to explore the application of these references in other clinical settings and to make useful comparisons with findings in uncomplicated DC and complicated MC twin pregnancies.

There is still a great deal more to understand about the physiopathology of Doppler velocimetry in MC twins, its quantitative and qualitative changes in the course of both complicated and uncomplicated gestations and the association with perinatal and long-term outcomes. Furthermore, the fascinating differences with singletons emphasise the biological uniqueness of these pregnancies, prompting further speculation on the specific interactions which take place both between the twins, and between the fetuses and the placenta. Clearly, the first step towards better understanding is the use of proper references, and the present study hopes to offer a useful contribution to this fundamental goal.

## Supporting information

S1 Table Percentage of observations below the 5^th^, 50^th^ and 95^th^ centiles by class of gestational age (GA). (PDF)
