# Supplementary material for: Longitudinal Doppler references for monochorionic twins and comparison with singletons
Source: PLoS One. 2019 Dec 6;14(12):e0226090. doi: 10.1371/journal.pone.0226090 (PMC6897428; doi:10.1371/journal.pone.0226090)
Supplement: S1 Table — (PDF) [file pone.0226090.s001.pdf]

S 1 Table. Percentage of observations below the 5th, 50th and 95th centiles by class of gestational age (GA).

Centile	GA class (complete week)						Total
	**<22**	**22-24**	**25-27**	**28-30**	**31-33**	**34+**	
UA							
05th	4.40	6.08	4.89	5.37	4.39	5.09	5.09
50th	51.60	48.91	46.74	50.70	48.29	50.40	49.33
95th	95.20	94.40	95.92	94.86	94.15	95.98	95.04
MCA PI							
05th	4.55	2.73	8.33	6.28	5.47	2.81	5.09
50th	57.85	57.07	48.61	51.93	52.08	46.88	52.28
95th	95.87	95.78	93.61	94.44	95.57	95.31	95.05
MCA PSV							
05th	6.64	3.47	4.71	5.29	6.99	3.75	5.08
50th	52.70	50.25	49.58	52.88	49.48	47.50	50.38
95th	94.61	95.54	96.40	93.51	94.30	95.94	95.02
DV PI							
05th	4.31	5.03	5.67	5.54	6.12	2.69	5.08
50th	47.84	50.26	51.94	49.34	52.77	47.98	51.26
95th	95.69	94.71	95.22	95.25	94.46	95.52	95.08
